# Associations Between Handgrip Strength and Markers of Insulin Resistance and Inflammation in Childhood and Adolescence: A Systematic Review With Meta‐Analysis

**DOI:** 10.1155/tsm2/1091342

**Published:** 2026-01-05

**Authors:** Takashi Abe, Ricardo B. Viana, Akemi Abe, Shuichi Machida, Hisashi Naito, Jeremy P. Loenneke

**Affiliations:** ^1^ Institute of Health and Sports Science & Medicine, Juntendo University, Inzai, Chiba, Japan, juntendo.ac.jp; ^2^ Division of Children’s Health and Exercise Research, Institute of Trainology, Fukuoka, Fukuoka, Japan; ^3^ Human Anatomy Laboratory, Institute of Physical Education and Sport, Federal University of Ceará, Fortaleza, Ceará, Brazil, ufc.br; ^4^ Graduate School of Health and Sports Science, Juntendo University, Inzai, Chiba, Japan, juntendo.ac.jp; ^5^ Department of Health, Exercise Science, and Recreation Management, Kevser Ermin Applied Physiology Laboratory, The University of Mississippi, Oxford, Mississippi, USA, olemiss.edu

**Keywords:** biomarkers, c-reactive protein, glucose metabolism, grip strength, pediatrics

## Abstract

**Background:**

Research on the association between changes in handgrip strength (HGS) and risk factors for lifestyle‐related diseases in children and adolescents may help clarify the inverse association between HGS and morbidity/mortality.

**Objective:**

This systematic review with meta‐analysis aimed to investigate the cross‐sectional and longitudinal associations between HGS and markers of insulin resistance and inflammation in children and adolescents.

**Methods:**

Observational studies that investigated the cross‐sectional and/or longitudinal associations between HGS and markers of insulin resistance and inflammation in children and adolescents were searched. Summary effect size measures were calculated using a random‐effects model estimation and reported as Fisher’s *r*‐to‐*z* transformed correlation coefficients and 95% confidence intervals.

**Results:**

Fifteen studies (12 cross‐sectional, two cross‐sectional and longitudinal, and one longitudinal) were included in the systematic review, of which 11 studies were also included in the meta‐analyses for cross‐sectional correlation. Relative (per body mass) but not absolute HGS was significantly associated (very low evidence) with markers of insulin resistance. Relative HGS was also significantly associated (very low evidence) with most of the inflammatory markers investigated. The three longitudinal studies included had insufficient information to perform a meta‐analysis.

**Conclusions:**

The results from cross‐sectional studies indicated the association (very low evidence) between HGS and several markers of insulin resistance and inflammation existed when studies utilized the relative HGS per body mass. However, no significant relationship was found when studies used absolute HGS. Furthermore, as longitudinal studies were limited, future longitudinal follow‐up studies are an important means of resolving these issues.

## 1. Introduction

Large‐scale longitudinal studies in middle‐aged and older adults have repeatedly reported inverse associations between handgrip strength (HGS) and the risk of diabetes [1, 2], heart disease [3, 4], cancer [5, 6], dementia [7, 8], and falls [9]. These associations remain even when adjusting for age, education level, body mass index, alcohol, tobacco, medical history, and others. Genetic [10] and nongenetic [11] factors have been proposed to explain these associations. However, what mechanisms explain the inverse association between HGS and morbidity/mortality remains unclear.

Although HGS is a biomarker [12], whether it can improve morbidity and mortality when increased by environmental factors such as sports and exercise training has yet to be demonstrated [13, 14]. Our recent studies revealed that HGS may increase through select sports (i.e., whether or not an athlete plays with sports equipment in their hands) during the period of development [15], and it is possible to affect HGS in young adulthood [16, 17]. The importance of HGS levels acquired during the developmental period is understandable, given that HGS, determined in early adulthood, changes significantly only when age‐related decline or injury/disease occurs [18, 19]. Furthermore, the HGS acquired during development may be associated with protection or resistance to developing lifestyle‐related disease risk factors, influencing morbidity and mortality throughout life.

Research in adults has shown a link between inflammatory markers [20] or insulin resistance [21] and mortality, with higher all‐cause mortality observed in groups with elevated risk factors. Additionally, individuals with higher levels of inflammatory markers tend to have weaker HGS in older adults [22]. These associations have also been shown in the follow‐up study reported inverse associations between baseline HGS and changes in inflammation markers in older women and speculated that inflammation markers partly explained the association between HGS and mortality [23]. A systematic review and meta‐analysis recently reported that higher levels of circulating inflammatory markers are significantly associated with lower muscle strength, including HGS in adults (≥ 18 years) [24]. However, the existence of similar associations between HGS and inflammatory markers or between HGS and insulin resistance in children and adolescents has not been systematically examined. Thus, this systematic review with meta‐analysis investigated the cross‐sectional and longitudinal associations between HGS and markers of insulin resistance and inflammation in children and adolescents using baseline values and/or change scores. Our hypotheses were that (i) there would be significant cross‐sectional associations between the HGS and insulin resistance and inflammatory markers, and (ii) there would be significant longitudinal associations between changes in HGS and changes in insulin resistance and inflammatory markers.

## 2. Methods

We performed this systematic review according to the Preferred Reporting Items for Systematic Review and Meta‐Analysis (PRISMA) statement [25]. The study was pre‐registered (February 3, 2024) in the International Prospective Register of Systematic Review (PROSPERO) (CRD42024502179).

### 2.1. Search Strategy

English‐language searches of the electronic databases Medical Literature Analysis and Retrieval System Online (MEDLINE/PubMed), Scopus, Web of Science, Excerpta Medica Database (Embase), and Cochrane Central Register of Controlled Trials (CENTRAL) were run by two independent researchers (T.A. and R.B.V.). Articles were retrieved from electronic databases combining the following terms: (handgrip strength OR grip strength OR grip) AND (resistin OR insulin resistance OR insulin sensitivity OR inflammation OR cytokines OR acute phase proteins) AND (child or children or ten or teenager or pediatric or adolescents or adolescence or juvenile). Supporting Information 1 shows the completed search strategy.

### 2.2. Eligibility Criteria

Observational studies examining the association between insulin resistance or inflammatory markers and HGS as a primary or secondary aim through any type of measurement and collected during childhood age (< 18 years) were included. Studies were excluded based on the following file types: study protocols, conference papers, letters to the editor, books, book sections, theses, film/broadcasts, case studies/reports, opinion articles, abstracts, or reviews. Rayyan software was used independently by two researchers (T.A. and R.B.V.) to remove duplicates and apply the eligibility criteria with disagreements resolved by a consensus between both researchers.

### 2.3. Data Extraction

The following study characteristics were extracted: authors, publication year, country, study design (cross‐sectional or longitudinal [cohort or case–control]), participant characteristics (age, body mass, height, body mass index, sex, and study sample), sample size, insulin resistance markers, inflammatory markers, HGS, effect size, technique used to measure insulin resistance, inflammation, and HGS, and information pertaining to methodological quality. In the event that the same participants are included across multiple articles, the study with the largest sample size and most comprehensive data extraction information was selected. If the same participants are included across multiple articles, but the available data are from different outcomes [e.g., fasting glucose, fasting insulin, homeostatic model assessment for insulin resistance (HOMA‐IR), and quantitative insulin sensitivity check index (QUICKI)], both effect sizes were extracted and examined in separate meta‐analyses. If a study analyzed the relationship between HGS and more than one insulin resistance marker or inflammatory marker concurrently, effect sizes for each association were calculated. These data were extracted independently by two researchers (T.A. and R.B.V.) with disagreements resolved by a consensus between both researchers.

### 2.4. Study Quality Assessment

Methodological quality of the included studies was assessed using the Joanna Briggs Critical Appraisal Tool [26]. Studies were assessed on an 8‐point (cross‐sectional), 10‐point (case–control), or 11‐point (cohort) scale. Each criterion was coded as “Yes” (1), “No” (0), “Unclear” (0), or “Not Applicable,” and a proportion of the total quality assessment score for each study was calculated based on the total number of items applicable to the study. Studies with a score higher than 70% were classified as having a high quality, those with a score between 50% and 70% as having a medium quality, and those with a score less than 50% as having a low quality. Two researchers (R.B.V. and T.A.) assessed the methodological quality.

### 2.5. Certainty of Evidence Assessment

Based on the Grading of Recommendations, Assessment, Development, and Evaluation (GRADE) method, one author (R.B.V.) rated the certainty for the main comparison and outcome as very low (very uncertainty about the estimate), low (research is very likely to significantly affect our confidence in estimating the effect and is likely to change the estimate), moderate (further research is likely to have an important impact on our confidence in estimating the effect and may change the estimate), or high (further research is very unlikely to change our confidence in estimating the effect) [27].

### 2.6. Statistical Analysis

For the cross‐sectional studies that only reported standardized beta (*β*) coefficients within the range from −0.50 to 0.50, correlation coefficient (*r*) values were calculated by the following equation: *r* = 0.98*β* + 0.05*λ* [28], where *λ* is an indicator variable that equals 1 when *λ* is nonnegative and 0 when *λ* is negative [28]. After obtaining *r* values, Fisher’s *Z* values for each cross‐sectional study were calculated from *r* values by the following equation: *Z* = 0.5 × ln((1 + *r*)/(1 − *r*)) [29], where ln(*x*) is the natural logarithm function. Standard error of Fisher’s *Z* values was calculated the following equation: ${\text{SE}}_{Z}=\sqrt{1/\left(n-3\right)}$ [29]. For the cross‐sectional studies that reported *r* values split by sex (boys and girls) or by age (children and youth), it was converted to Fisher’s *Z* values and combined to a single value (effect), as recommended by Borenstein et al. [29]. Correlation meta‐analyses using Fisher’s *r*‐to‐*z* transformed correlation coefficients were performed to determine the overall correlation between HGS (absolute and relativized by body mass) and insulin resistance and inflammatory markers. For that, Fisher’s *Z* and its respective standard error values were pooled under a random‐effects model. The results, such as the summary effect and its confidence interval, were then converted back to *r* values for presentation [29]. The *r* values were interpreted per Pearson thresholds [30]: trivial (< 0.10), small (0.10 to < 0.30), moderate (0.30 to < 0.50), and large (≥ 0.5). A random‐effects model was used to reduce the risk of unknown factors responsible for variability even under homogeneity. Restricted maximum likelihood estimation was used in all models. To improve our results, we conducted several sensitivity analyses (the one study removed method) to consider the influence of each study on the overall results. Due to insufficient longitudinal included studies and available information, it was not possible to pooled data for associations between changes in HGS and the changes in insulin resistance and inflammatory markers.

Statistical heterogeneity was assessed using *τ* ^2^, *H* ^2^, *Q* statistic, and the inconsistency *I* ^2^ test. The *I* ^2^ statistic estimates the percentage variance between studies and can be roughly interpreted as low (0%–40%), moderate (30%–60%), substantial (50%–90%), or considerable (75%–100%) heterogeneity. To note, the *I* ^2^ classifications overlap as these are rough guidelines suggested by Higgins et al. [31]. Publication bias was visually assessed using funnel plots by plotting the effect size of each trial against its standard error. As recommended by Higgins et al. [31], “Egger’s regression test” was not performed to assess asymmetry of the funnel plot because all between‐groups meta‐analyses involved less than 10 original studies. All statistical analyses were performed in Jeffreys’s Amazing Statistics Program (JASP, 0.18.3.0, Netherlands) using an alpha level of *p* < 0.05 [32]. All statistical analyses were performed using an alpha level of *p* ≤ 0.05.

## 3. Results

### 3.1. Included Studies

**Figure 1:**
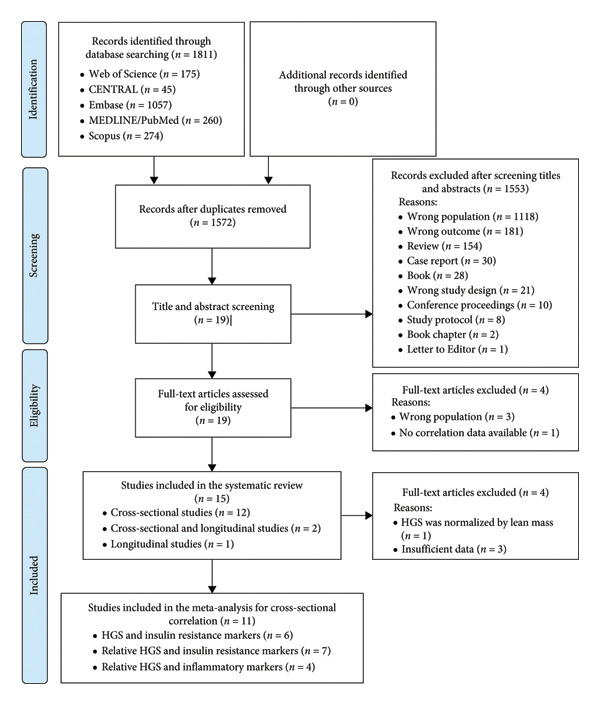
Study flow diagram of the selection process for the studies included in this systematic review and meta‐analysis. *n* indicates the number of studies. CENTRAL: Cochrane Central Register of Controlled Trials. Embase: Excerpta Medica Database. MEDLINE/PubMed: Medical Literature Analysis and Retrieval System Online. HGS: handgrip strength.

The search strategy retrieved 1811 records (Embase [*n* = 1057], CENTRAL [*n* = 45], MEDLINE [*n* = 260], Scopus [*n* = 274], and Web of Science [*n* = 175]). After duplications were removed (*n* = 239), title and abstract of 1572 records were screened and 1553 records were eliminated due to the following reasons: wrong population (*n* = 1118), wrong outcome (*n* = 181), review (*n* = 154), case report (*n* = 30), book (*n* = 28), wrong study, design (*n* = 21), conference proceedings (*n* = 10), study protocol (*n* = 8), book chapter (*n* = 2), and letter to editor (*n* = 1). The remaining 19 full‐text articles were reviewed further, with four studies excluded due the following reasons: wrong population (*n* = 3) and no correlation data available for HGS and insulin resistance or inflammatory markers (*n* = 1) (Supporting Information 2). Thus, 15 studies were included in this systematic review (12 cross‐sectional [33–44], two cross‐sectional and longitudinal [45, 46], and one longitudinal [44, 47]). As eight [36, 39, 41–46] of the 14 cross‐sectional included studies did not report *r* values for the cross‐sectional correlation between HGS and inflammatory and/or insulin resistance markers, this information was requested for the corresponding authors. However, only four of the corresponding authors sent the data requested [36, 41, 44, 45], one author answered that the raw data were lost and informed that B coefficients were unstandardized [43], one author declined our request [42], and the remaining two authors did not respond to our request [39, 46]. Thus, the *r* values for one [46] of the two studies [39, 46] that reported standardized *β* coefficients within the range from −0.50 to 0.50 for the cross‐sectional analyses were estimated as previously reported in the statistical analysis section. Three studies [42, 43, 47] were excluded from the meta‐analysis because it was not possible to estimate *r* values, and one study [39] was excluded because HGS was relativized by lean mass (Supporting Information 2). Therefore, 11 of the 15 included studies were also included in the meta‐analysis for cross‐sectional correlation (eight for insulin resistance [35–37, 40, 41, 44–46] and four for inflammation [33, 34, 37, 38] (Figure 1). The included studies were published from 2011 [35] up to 2023 [39] (Table 1).

**Table 1:** Characteristics of the participants of the included studies (*n* = 15).

Study	Study design	Study sample	Sex (*n*)	Age (years)^a^	Body mass (kg)^a^	Height (cm)^a^	BMI (kg/m^2^)^a^
Agostinis‐Sobrinho et al. [33]	Cross‐sectional	LabMed physical activity study. Adolescents from schools in four Portuguese cities	Boys (*n* = 262) Girls (*n* = 267)	14.39 ± 1.74 14.27 ± 1.71	56.89 ± 14.10 53.44 ± 11.18	162.9 ± 11.3 157.7 ± 6.68	21.20 ± 3.73 21.41 ± 3.96

Artero et al. [35]	Cross‐sectional	HELENA‐CSS. Adolescents’ students from 10 European cities from nine countries	Boys (*n* = 346) Girls (*n* = 363)	14.9 ± 1.3 14.9 ± 1.2	62.0 ± 13.9 56.1 ± 10.7	170.8 ± 9.7 162.0 ± 7.2	21.1 ± 3.7 21.3 ± 3.4

Artero et al. [34]	Cross‐sectional	HELENA‐CSS. Adolescents’ students from 10 European cities from nine countries	Boys (*n* = 296) Girls (*n* = 343)	14.8 ± 1.3 14.9 ± 1.3	60.9 ± 13.5 55.9 ± 10.6	170.2 ± 10.0 162.2 ± 7.1	20.9 ± 3.6 21.2 ± 3.4

Castro‐Piñero et al. [36]	Cross‐sectional	HELENA‐CSS. Adolescents’ students from 10 European cities from nine countries	Boys (*n* = 444) Girls (*n* = 506)	14.7 ± 1.2 14.7 ± 1.2	61.3 ± 13.7 55.6 ± 10.2	169.3 ± 9.8 161.7 ± 7.1	NR NR

Cohen et al. [37]	Cross‐sectional	ACFIES. Low‐middle socioeconomic status schoolchildren enrolled in public elementary and high schools in Bucaramanga, Colombia	Boys (*n* = 351) Girls (*n* = 318)	11.51 ± 1.16 11.52 ± 1.10	39.86 ± 10.35 40.33 ± 9.77	144 ± 9 146 ± 8	18.93 ± 3.68 18.81 ± 3.52

Delgado‐Alfonso et al. [38]	Cross‐sectional	UP&DOWN. Children and adolescents from schools in Cadiz and Madrid, Spain	Children (*n* = 230) Youth (*n* = 273)	8.1 ± 1.5 14.1 ± 1.6	30.7 ± 9.6 54.8 ± 12.8	129.1 ± 11.1 160.4 ± 10.5	NR NR

Demmer et al. [47]	Longitudinal (cohort)	Raine. Adolescents living in Perth, Australia	10 years: Boys (*n* = 849) 10 years: Girls (*n* = 783) 14 years: Boys (*n* = 815) 14 years: Girls (*n* = 774) 17 years: Boys (*n* = 628) 17 years: Girls (*n* = 616)	NR NR NR NR NR NR	38.57 ± NR 38.71 ± NR 58.62 ± NR 56.67 ± NR 72.29 ± NR 63.66 ± NR	143.69 ± NR 143.64 ± NR 166.27 ± NR 162.02 ± NR 178.3 ± NR 165.9 ± NR	20.97 ± NR NR 21.06 ± NR 21.53 ± NR 22.69 ± NR 23.12 ± NR

Haapala et al. [39]	Cross‐sectional	PANIC. Primary school children in Kuopio, Finland	Boys (*n* = 202) Girls (*n* = 189)	7.7 ± 0.4 7.6 ± 0.4	26.6 ± 5.5 25.6 ± 5.9	129.7 ± 5.2 127.7 ± 5.8	NR NR

Jimenez‐Pavon et al. [40]	Cross‐sectional	HELENA‐CSS. Adolescents’ students from 10 European cities from nine countries	Boys (*n* = 499) Girls (*n* = 554)	14.9 ± 1.3 14.9 ± 1.2	62.1 ± 14.0 56.0 ± 10.2	170 ± 10 160 ± 10	21.4 ± 3.8 21.3 ± 3.4

Jung et al. [41]	Cross‐sectional	KNHANES. Korean children and youth	Boys (*n* = 1487) Girls (*n* = 1310)	14.4 ± 0.1^b^ 14.4 ± 0.1^b^	Overall weight *z*‐score: 0.4 ± 0.0^b^	Overall height *z*‐score: 0.6 ± 0.0^b^	Overall BMI *z*‐score: 0.2 ± 0.0^b^

Lang et al. [42]	Cross‐sectional	CHMS. Canadians’ children and youth	Children boy (*n* = 1086) Children girl (*n* = 1071) Youth boy (*n* = 857) Youth girl (*n* = 786)	8.5 ± NR 8.7 ± NR 14.4 ± NR 14.6 ± NR	NR NR NR NR	NR NR NR NR	18.2 ± NR 17.7 ± NR 21.9 ± NR 22.1 ± NR

Li et al. [43]	Cross‐sectional	NHANES. Children in the non‐institutionalized general population of the United States	Boys (*n* = 474) Girls (*n* = 485)	15.3 ± 2.3 15.4 ± 2.3	NR NR	NR NR	23.9 ± 6.0 24.3 ± 6.7

López‐Gil et al. [44]	Cross‐sectional (cohort)	Children enrolled in the Growth and Obesity Chilean Cohort Study	Boys (*n* = 185) Girls (*n* = 267)	7.8 ± 0.5 7.9 ± 0.4	27.9 [7.6]^c^ 28.2 [8.1]^c^	126.6 ± 5.6 127.1 ± 5.8	17.1 [3.2]^c^ 17.4 [3.9]^c^

Tarp et al. [45]	Cross‐sectional and longitudinal (cohort)	CHAMPS‐study DK. Public school children in Svendborg, Denmark	Boys (*n* = 253) Girls (*n* = 259)	8.5 ± 1.4 8.4 ± 1.4	28.6 27.8	134.4 ± 10.4 132.0 ± 9.4	NR NR

Zaqout et al. [46]	Cross‐sectional and longitudinal (cohort)	IDEFICS. Children from eight European countries	Boys (*n* = 786) Girls (*n* = 770)	Overall: 8.4 ± 1.6^d^	NR	NR	Overall BMI *z*‐score: 0.21 ± 1.31

*Note:* LabMed Physical Activity Study: Longitudinal Analysis of Biomarkers and Environmental Determinants of Physical activity. HELENA‐CSS: Healthy Lifestyle in Europe by Nutrition in Adolescence Cross‐sectional study. ACFIES: Association between Cardiorespiratory Fitness, Muscular Strength and Body Composition with Metabolic Risk Factors in Colombian Children. UP&DOWN: Follow‐up in healthy schoolchildren and in adolescents with DOWN syndrome. Raine: Western Australian Pregnancy Cohort study. CDAH: Childhood Determinants of Adult Health study. PANIC: Physical Activity and Nutrition in Children study. CHAMPS‐study DK: Childhood Health, Activity and Motor Performance School Study Denmark. IDEFICS: IDentification and prevention of dietary and lifestyle induced health EFfects In Children and infantS. BMI_*z*_‐score: age and sex adjusted BMI_*z*_‐scores.

Abbreviations: BMI = body mass index, CHMS = Canadian Health Measures Survey, KNHANES = Korea National Health and Nutrition Examination Survey, NHANES = National Health and Nutrition Examination Survey, NR = not reported.

^a^Data presented as mean ± standard deviation.

^b^Data presented as mean ± standard error.

^c^Data presented as median [interquartile range].

^d^Age regarding baseline sample (*n* = 1635).

### 3.2. Participant Characteristics

Participants’ characteristics are summarized in Table 1. Almost one‐third of the included studies (*n* = 6) were conducted with adolescents [33–36, 40, 43], while the other four studies were conducted with children [39, 44–46], and five were mixed children and adolescents [37, 38, 41, 42, 47]. The age range of the samples in many studies was between five and 8 years [30–34, 37, 38, 40, 43, 44], although the range was narrower for studies of children [39, 44, 45] and wider for mixed children and adolescents [38, 42]. The study sample was collected from Europe [33–36, 38–40], North America [42, 43], South America [37, 44], Asian [41] countries, and Australia [47]. Five studies reported the prevalence of overweight and obesity [34, 35, 37, 39, 41], while other studies did not clearly describe them.

### 3.3. HGS Assessment

Electronic digital or analog hand dynamometers were used for measuring HGS in 11 studies for meta‐analysis [33–38, 40, 41, 44–46]. Almost half of these studies (*n* = 6) did not clearly report which posture (e.g., standing or sitting) was adopted for HGS measurements [34–36, 38, 40, 44], but five other studies performed measurements in the standing position [33, 37, 41, 45, 46]. In almost all studies included in the meta‐analyses [33–38, 40, 45, 46], the grip span was adjusted to participants’ hand size.

### 3.4. Meta‐Analyses: HGS and Insulin Resistance Markers

The available data from the included studies (Table 2) allowed us to conduct correlation meta‐analyses pooling Fisher’s *r*‐to‐*z* transformed correlation coefficients for absolute HGS and fasting glucose, fasting insulin, and HOMA‐IR, as well as for relative HGS (relativized by body mass) and these same insulin resistance markers. All meta‐analyses pooling Fisher’s *r*‐to‐*z* transformed correlation coefficients are summarized in Table 3.

**Table 2:** Characteristics of the outcome measurements and main results of the included studies (*n* = 15).

Study	Study design	*N*	%boys	Markers	Correlations (*p* value)	Comments
Agostinis‐Sobrinho et al. [33]	Cross‐sectional (cohort)	529	49.5	Leptin	*r* = −0.462 (*p* < 0.001) (relative HGS)	HGS was the relative value of dividing by body mass. The *r* values were adjusted for age, sex, and pubertal stage
				hs‐CRP	*r* = −0.243 (*p* < 0.001) (relative HGS)	
				C3	*r* = −0.245 (*p* < 0.001) (relative HGS)	
				C4	*r* = −0.140 (*p* < 0.001) (relative HGS)	
				Fibrinogen	*r* = −0.172 (*p* < 0.001) (relative HGS)	
				IL‐6	*r* = 0.017 (NS) (relative HGS)	
				Albumin	*r* = 0.016 (NS) (relative HGS)	
				Interferon‐α	*r* = 0.046 (NS) (relative HGS)	

Artero et al. [35]	Cross‐sectional (cohort)	709	48.8	HOMA‐IR	*r* = −0.186 (*p* < 0.001) (relative HGS)	HGS was the relative value of dividing by body mass. The *r* values were adjusted for age, sex, pubertal stage, and center

Artero et al. [34]	Cross‐sectional (cohort)	639	46.3	C3	*r* = −0.240 (*p* < 0.001) (relative HGS)	HGS was the relative value of dividing by body mass. The values were adjusted for age, sex, pubertal stage, and center (dummy variable)
				C4	*r* = −0.195 (*p* < 0.001) (relative HGS)	
				CRP	*r* = −0.139 (*p* < 0.001) (relative HGS)	

Castro‐Piñero et al. [36]	Cross‐sectional (cohort)	950	46.7	Fasting glucose	*r* = 0.105 (*p* = 0.001, overall) (absolute HGS)^a^	Absolute averaged left and right HGS was reported as well as it was divided by body mass. These results were sent by the authors and are crude data (not adjusted for age and/or sex)
					*r* = 0.013 (*p* = 0.793, boys) (absolute HGS)^a^	
					*r* = −0.105 (*p* = 0.018, girls) (absolute HGS)^a^	
				Fasting insulin	*r* = −0.170 (*p* < 0.001, overall) (absolute HGS)^a^	
					*r* = 0.042 (*p* = 0.374, boys) (absolute HGS)^a^	
					*r* = −0.171 (*p* < 0.001, girls) (absolute HGS)^a^	

Cohen et al. [37]	Cross‐sectional (cohort)	669	47.5	Fasting glucose	*r* = −0.028 (NS) (relative HGS)	HGS was the relative value of dividing by body mass. The values were adjusted for age, sex, and maturation status and physical activity
				HOMA‐IR	*r* = −0.1775 (*p* < 0.001) (relative HGS)	
				hs‐CRP	*r* = −0.107 (*p* < 0.05) (relative HGS)	

Delgado‐Alfonso et al. [38]	Cross‐sectional (cohort)	Children: 230 Youth: 273		CRP	*r* = −0.268 (*p* < 0.010, children) (relative HGS)	HGS was the relative value of dividing by body mass. The values were adjusted for sex and maturation status (Tanner stage) and body mass index
					*r* = −0.173 (*p* < 0.050, youth) (relative HGS)	
				TNF‐α	*r* = −0.091 (NS, children) (relative HGS)	
					*r* = 0.012 (NS, youth) (relative HGS)	
				C3	*r* = −0.173 (*p* < 0.010, children) (relative HGS)	
					*r* = −0.180 (*p* < 0.010, youth) (relative HGS)	
				C4	*r* = −0.214 (*p* < 0.010, children) (relative HGS)	
					*r* = −0.137 (*p* < 0.050, youth) (relative HGS)	
				IL‐6	*r* = −0.247 (*p* < 0.001, children) (relative HGS)	
					*r* = −0.103 (NS, youth) (relative HGS)	

Demmer et al. [47]	Longitudinal (cohort)	14 years: 1346	14 years: 51.3	HOMA‐IR hs‐CRP	*β* = 0.003 (*p* = 0.013) (adjusted HGS)^b^	It was calculated a height‐adjusted and weight‐adjusted HGS. The results reported unadjusted and adjusted by body mass index. The results were positive when unadjusted and negative after adjustment
		17 years: 1048	17 years: 50.5		*β* = 0.005 (*p* = 0.013) (adjusted HGS)^b^	

Haapala et al. [39]	Cross‐sectional (cohort)	391	51.7	hs‐CRP	*β* = 0.002 (NS) (relative HGS)^c^ *r* = 0.052 (NS) (relative HGS)^d^	HGS was relative value of dividing by lean mass. The results were adjusted for age and sex, or age, sex and %fat, or age, sex, and body mass index–standard deviation score
				Leptin	*β* = −0.026 (NS) (relative HGS)^c^ *r* = −0.025 (NS) (relative HGS)^d^	
				Leptin receptor	*β* = 0.047 (NS) (relative HGS)^c^ *r* = 0.096 (NS) (relative HGS)^d^	
				HMW‐adiponectin	*β* = −0.043 (NS) (relative HGS)^c^ *r* = −0.042 (NS) (relative HGS)^d^	
				IL‐6	*β* = −0.030 (NS) (relative HGS)^c^ *r* = −0.029 (NS) (relative HGS)^d^	
				TNF‐α	*β* = −0.073 (NS) (relative HGS)^c^ *r* = −0.072 (NS) (relative HGS)^d^	
				GlycA	*β* = −0.069 (NS) (relative HGS)^c^ *r* = −0.068 (NS) (relative HGS)^d^	

Jimenez‐Pavon et al. [40]	Cross‐sectional (cohort)	1053	47.4	Fasting glucose	*r* = −0.006 (boys) (absolute HGS)	Absolute combined left and right HGS was reported, and it was divided by body mass. These are the results from partial correlations, controlled by pubertal status. Multiple linear regression shows significant associations
					*r* = −0.085 (NS) (girls) (absolute HGS)	
					*r* = 0.004 (boys) (relative HGS)	
					*r* = −0.062 (girls) (relative HGS)	
				Fasting insulin	*r* = 0.071 (NS, boys) (absolute HGS)	
					*r* = −0.011 (NS, girls) (absolute HGS)	
					*r* = −0.249 (boys) (relative HGS)	
					*r* = −0.205 (girls) (relative HGS)	
				HOMA‐IR	*r* = 0.058 (boys) (absolute HGS)	
					*r* = −0.016 (NS, girls) (absolute HGS)	
					*r* = −0.244 (boys) (relative HGS)	
					*r* = −0.213 (girls) (relative HGS)	
				QUICKI	*r* = −0.075 (boys) (absolute HGS)	
					*r* = 0.028 (NS, girls) (absolute HGS)	
					*r* = 0.230 (boys) (relative HGS)	
					*r* = 0.188 (girls) (relative HGS)	

Jung et al. [41]	Cross‐sectional (cohort)	2797	53.2	Fasting glucose	*r* = −0.0115 (NR, absolute HGS)^a^	Absolute combined left and right HGS was reported, and it was divided by body mass. These results were sent by the authors and are crude data (not adjusted for age and/or sex)
					*r* = −0.0615 (NR, relative HGS)^a^	
				Fasting insulin	*r* = −0.0162 (NR, absolute HGS)^a^	
					*r* = −0.3278 (NR, relative HGS)^a^	
				HOMA‐IR	*r* = −0.0188 (NR, absolute HGS)^a^	
					*r* = −0.3232 (NR, relative HGS)^a^	
				HbA1c	*r* = −0.0222 (NR, absolute HGS)^a^	
					*r* = −0.0357 (NR, relative HGS)^a^	
				Insulin resistance	OR = 0.026 (*p* < 0.01) (relative HGS)	

Lang et al. [42]	Cross‐sectional (cohort)	Children boy: 1086 Children girl: 1071 Youth boy: 857 Youth girl: 786	Children: 50.3 Youth: 52.2	CRP	*B* = 0.1 (children boy) (adjusted HGS)^e^	Absolute combined left and right HGS was converted to age‐ and sex‐standardized z‐scores for statistical analysis
					*B* = −0.1 (children girl) (adjusted HGS)^e^	
					*B* = −0.1 (youth boy) (adjusted HGS)^e^	
					*B* = −0.0 (youth girl) (adjusted HGS)^e^	
				Nonfasted glucose	*B* = −0.0 (children boy) (adjusted HGS)^e^	
					*B* = 0.0 (children girl) (adjusted HGS)^e^	
					*B* = 0.0 (youth boy) (adjusted HGS)^e^	
					*B* = 0.0 (youth girl) (adjusted HGS)^e^	
				HbA1c	*B* = −0.0 (children boy) (adjusted HGS)^e^	
					*B* = −0.0 (children girl) (adjusted HGS)^e^	
					*B* = 0.0 (youth boy) (adjusted HGS)^e^	
					*B* = 0.0 (youth girl) (adjusted HGS)^e^	

Li et al. [43]	Cross‐sectional (cohort)	959	49.4	Fasting glucose	*B* = 0.01 (*p* = 0.77, overall) (relative HGS)^e^	Absolute combined left and right HGS was reported, and it was divided by body mass. Results were presented only for relative HGS adjusted for age, race, body mass index, and physical activity
					*B* = 0.01 (*p* = 0.82, boys) (relative HGS)^e^	
					*B* = 0.02 (*p* = 0.54, girls) (relative HGS)^e^	
				Fasting insulin	*B* = −0.003 (*p* = 0.017, overall) (relative HGS)^e^	
					*B* = −0.005 (*p* = 0.001, boys) (relative HGS)^e^	
					*B* = −0.002 (*p* = 0.45, girls) (relative HGS)^e^	
				HOMA‐IR	*B* = −0.003 (*p* = 0.025, overall) (relative HGS)^e^	
					*B* = −0.005 (*p* = 0.002, boys) (relative HGS)^e^	
					*B* = −0.002 (*p* = 0.52, girls) (relative HGS)^e^	
				2 h glucose	*B* = −0.27 (*p* < 0.0001, overall) (relative HGS)^e^	
					*B* = −0.27 (*p* = 0.0006, boys) (relative HGS)^e^	
					*B* = −0.23 (*p* = 0.09, girls) (relative HGS)^e^	

López‐Gil et al. [44]	Cross‐sectional (cohort)	452	40.9%	Fasting glucose	*r = *−0.033 (*p* = 0.479, overall) (absolute HGS)^a^	Absolute average of left and right HGS values was reported, and it was divided by body mass
					*r = *−0.086 (*p* = 0.243, boys) (absolute HGS)^a^	
					*r = *0.017 (*p* = 0.781, girls) (absolute HGS)^a^	
					*r = *−0.138 (*p* = 0.003, overall) (relative HGS)^a^	
					*r = *−0.223 (*p* = 0.002, boys) (relative HGS)^a^	
					*r = *−0.080 (*p* = 0.194, girls) (relative HGS)^a^	
				Fasting insulin	*r = *0.022 (*p* = 0.636, overall) (absolute HGS)^a^	
					*r = *0.036 (*p* = 0.629, boys) (absolute HGS)^a^	
					*r = *0.009 (*p* = 0.884, girls) (absolute HGS)^a^	
					*r = *−0.153 (*p* = 0.001, overall) (relative HGS)^a^	
					*r = *−0.101 (*p* = 0.170, boys) (relative HGS)^a^	
					*r = *−0.185 (*p* = 0.002, girls) (relative HGS)^a^	
				HOMA‐IR	*r = *0.006 (*p* = 0.895, overall) (absolute HGS)^a^	
					*r = *−0.002 (*p* = 0.977, boys) (absolute HGS)^a^	
					*r = *0.005 (*p* = 0.936, girls) (absolute HGS)^a^	
					*r = *−0.179 (*p* < 0.001, overall) (relative HGS)^a^	
					*r = *−0.174 (*p* = 0.018, boys) (relative HGS)^a^	
					*r = *−0.191 (*p* = 0.002, girls) (relative HGS)^a^	

Tarp et al. [45]	Cross‐sectional and longitudinal (case control)	512	49.4	HOMA‐IR	Cross‐sectional: *r* = 0.3392 (*p* = 0.068, boys) (absolute HGS)^a^	HGS was reported as the average of left and right maximum scores, and it was divided by body mass. Model included age, sex, school type, family history of cardiovascular disease, diabetes or hypertension, sexual maturity, mother’s body mass index, and mother’s educational attainment. However, the *r* values were sent by the authors and are crude data (not adjusted for age and/or sex)
					*r* = 0.3410 (*p* = 0.168, girls) (absolute HGS)^a^	
					*r* = −0.0903 (*p* = 0.068, boys) (relative HGS)^a^	
					*r* = −0.1791 (*p* = 0.168, girls) (relative HGS)^a^	
					Longitudinal: *β* = −0.06 (*p* = 0.17, overall) (relative HGS)^c^	

Zaqout et al. [46]	Cross‐sectional and longitudinal (case control)	1556	50.5	HOMA‐IR	Cross‐sectional: *β* = 0.040 (*p* = 0.030, overall) (absolute HGS)^c^	HGS was reported as the average of left and right maximum scores. Mixed‐models regression adjusted for sex, age, level of parental educational attainment, sugar and fat propensity score, and body mass index
					*r* = 0.0892 (overall) (absolute HGS)^d^	
					*β* = 0.045 (*p* = 0.068, boys) (absolute HGS)^c^	
					*β* = 0.039 (*p* = 0.168, girls) (absolute HGS)^c^	
					Longitudinal: *β* = −0.019 (*p* = 0.457, overall) (absolute HGS)^c^	
					*β* = −0.017 (*p* = 0.607, boys) (absolute HGS)^c^	
					*β* = −0.024 (*p* = 0.528, girls) (absolute HGS)^c^	

*Note:* HGS: handgrip strength. HOMA‐IR: homeostasis model assessment‐estimated insulin resistance. QUICKI: quantitative insulin sensitivity check index. HbA1c: glycated hemoglobin. C3: complement component 3. C4: complement component 4. IL‐6: interleukin‐6. GlycA: glycoprotein acetyls. lnOR: natural logarithm of the odds ratio.

Abbreviations: BMI = body mass index, CRP = C‐reactive protein, HMW‐adiponectin = high‐molecular weight adiponectin, hs‐CRP = high‐sensitivity C‐reactive protein, NS = not significant, OR = odds ratio, TNF‐α = tumor necrosis factor alpha.

^a^Data sent by the corresponding author.

^b^The authors did not report if beta coefficients were standardized or unstandardized.

^c^Standardized beta coefficient.

^d^Data calculated from standardized beta coefficient.

^e^Unstandardized beta coefficient.

**Table 3:** Synthesis of the meta‐analyses pooling Fisher’s *r*‐to‐*z* transformed correlation coefficients from the cross‐sectional included studies.

Correlation analysis		*k*	*c*	*n*	Summary effect			*p*	Heterogeneity			
Variable 1 (“predictor”)	Variable 2 (“outcome”)				Fisher’s *z* [95% CI]	*r* [95% CI]^a^	Classification^b^		*τ* ^2^	*I* ^2^ (%)	*H* ^2^	*p* (*Q* statistics)
HGS	Fasting glucose	4	4	5252	0.004 [–0.06 to 0.07]	0.004 [–0.06 to 0.07]	Trivial	0.902	0.004	80.6	5.2	0.003
HGS	Fasting insulin	3	3	4302	0.0008 [–0.03 to 0.03]	0.0008 [–0.03 to 0.03]	Trivial	0.962	0.0001	13.0	1.2	0.419
HGS	HOMA‐IR	5	5	6370	0.09 [–0.04 to 0.22]	0.09 [–0.04 to 0.22]	Trivial	0.190	0.021	96.0	25.1	< 0.001
HGS/BW	Fasting glucose	4	4	4971	−0.06 [–0.09 to −0.03]	−0.06 [–0.09 to −0.03]	Trivial	< 0.001	0.000004	0.4	1.0	0.219
HGS/BW	Fasting insulin	4	4	5252	−0.23 [–0.31 to −0.14]	−0.23 [–0.30 to −0.14]	Small	< 0.001	0.006	87.8	8.2	< 0.001
HGS/BW	HOMA‐IR	6	6	6192	−0.22 [–0.28 to −0.15]	−0.22 [–0.27 to −0.15]	Small	< 0.001	0.004	80.0	5.0	< 0.001
HGS/BW	CRP	2	2	1142	−0.18 [–0.26 to −0.10]	−0.18 [–0.25 to −0.10]	Small	< 0.001	0.001	44.7	1.8	0.179
HGS/BW	hs‐CRP	2	2	1198	−0.18 [–0.31 to −0.04]	−0.18 [–0.30 to −0.04]	Small	0.013	0.008	86.6	5.7	0.016
HGS/BW	C3	3	2	1671	−0.23 [–0.28 to −0.18]	−0.23 [–0.27 to −0.18]	Small	< 0.001	0.000	0.0	1.0	0.446
HGS/BW	C4	3	3	1671	−0.17 [–0.22 to −0.12]	−0.17 [–0.22 to −0.12]	Small	< 0.001	0.000	0.0	1.0	0.631
HGS/BW	IL‐6	2	2	1032	−0.08 [–0.26 to 0.11]	−0.08 [–0.25 to 0.11]	Trivial	0.413	0.016	89.0	9.1	0.003

*Note: k*: number of individual studies. *c*: number of independent comparisons. *n*: sample size. *r*: correlation coefficient. HGS: handgrip strength. HGS/BW: handgrip strength relativized by participants body weight. HOMA‐IR: homeostasis model assessment‐estimated insulin resistance. QUICKI: quantitative insulin sensitivity check index. C3: complement component 3. C4: complement component 4. IL‐6: interleukin‐6.

Abbreviations: CI = confidence interval, CRP = C‐reactive protein, hs‐CRP = high‐sensitivity C‐reactive protein.

^a^The summary effect and its confidence interval were converted back to *r* values for presentation and respective classification.

^b^The *r* values were interpreted per Pearson thresholds [28]: trivial (< 0.10), small (0.10 to < 0.30), moderate (0.30 to < 0.50), and large (≥ 0.5).

#### 3.4.1. Fasting Glucose

Four studies [36, 40, 41, 44] with four independent comparisons provided data from 5252 children and youth individuals to analyze the relationship between absolute HGS and fasting glucose. There was no significant correlation (*k* = 4; *c* = 4; *n* = 5252; Fisher’s *z* = 0.004; 95% CI = −0.06 to 0.07, *p* = 0.902) between absolute HGS and fasting glucose (Figure 2(a)) with considerable heterogeneity (*τ* ^2^ = 0.004, *I* ^2^ = 80.6%, *H* ^2^ = 5.2, *Q* [3] = 13.951, *p* = 0.003).

Figure 2Association between HGS and insulin resistance‐related variables. (a) The top panel is HGS and fasting glucose, (b) the middle panel is HGS and fasting insulin, and (c) the bottom panel represents HGS and HOMA‐IR. The black box represents study’s effect size, and the box size reflects study’s relative weight. The continuous black line represents the study’s 95% CI. The black diamond represents the aggregate effect size and 95% CI. Fisher’s *Z*: Fisher’s *r*‐to‐*z* transformed correlation coefficients. CI: confidence interval. HGS: handgrip strength. HOMA‐IR: model assessment‐estimated insulin resistance. REML: restricted maximum likelihood estimation.

**Figure:**
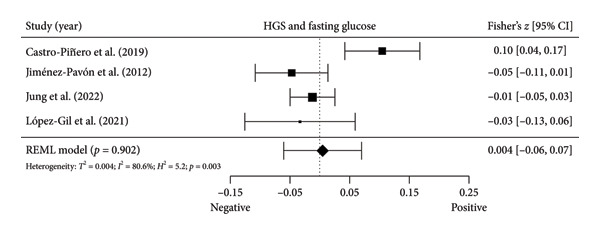
(a)

**Figure:**
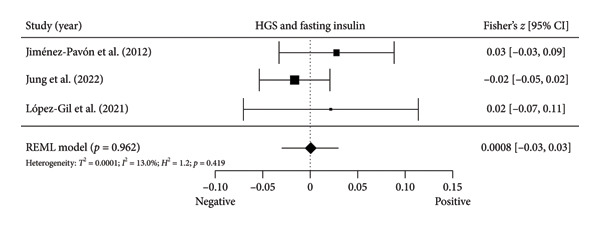
(b)

**Figure:**
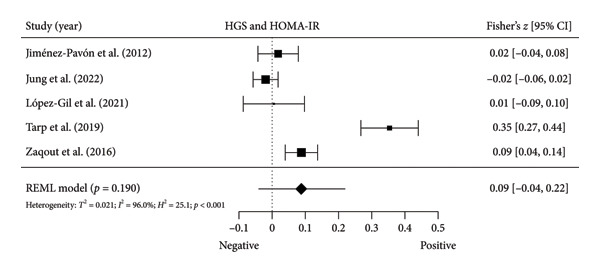
(c)

Four studies [37, 40, 41, 44] with four independent comparisons provided data from 4971 children and youth individuals to analyze the relationship between relative HGS and fasting glucose. There was a significant negative correlation (*k* = 4; *c* = 4; *n* = 4971; Fisher’s *z* = −0.06; 95% CI = −0.09 to −0.03, *p* < 0.001) between relative HGS and fasting glucose (Figure 3(a)) with low heterogeneity (*τ* ^2^ = 0.000004, *I* ^2^ = 0.4%, *H* ^2^ = 1.0, *Q* [4] = 4.422, *p* = 0.219).

Figure 3Association between relative HGS and insulin resistance markers. (a) The top panel is HGS/BW and fasting glucose, (b) the middle panel is HGS/BW and fasting insulin, and (c) the bottom panel represents HGS/BW and HOMA‐IR. The continuous black line represents the study’s 95% CI. The black diamond represents the aggregate effect size and 95% CI. Fisher’s *Z*: Fisher’s *r*‐to‐*z* transformed correlation coefficients. CI: confidence interval. HGS/BW: handgrip strength relativized by body mass. HOMA‐IR: model assessment‐estimated insulin resistance, REML: restricted maximum likelihood estimation.

**Figure:**
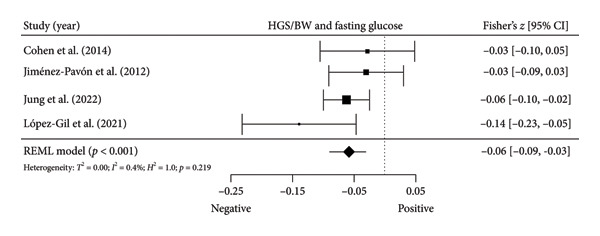
(a)

**Figure:**
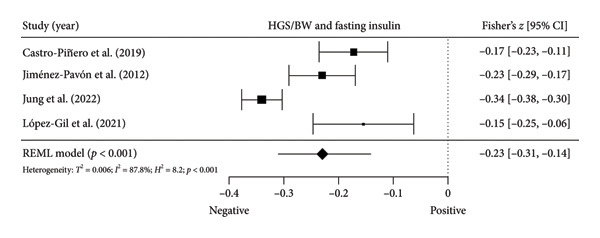
(b)

**Figure:**
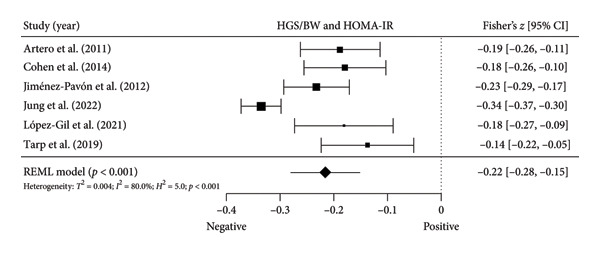
(c)

#### 3.4.2. Fasting Insulin

Three studies [40, 41, 44] with three independent comparisons provided data from 4302 children and youth individuals to analyze the relationship between absolute HGS and fasting insulin. There was no significant correlation (*k* = 3; *c* = 3; *n* = 4302; Fisher’s *z* = 0.0008; 95% CI = −0.03 to 0.03, *p* = 0.962) between absolute HGS and fasting glucose (Figure 2(b)) with low heterogeneity (*τ* ^2^ = 0.0001, *I* ^2^ = 13.0%, *H* ^2^ = 1.2, *Q* [2] = 1.738, *p* = 0.419).

Four studies [36, 40, 41, 44] with four independent comparisons provided data from 5252 children and youth individuals to analyze the relationship between relative HGS and fasting insulin. There was a significant negative correlation (*k* = 4; *c* = 4; *n* = 5252; Fisher’s *z* = −0.23; 95% CI = −0.31 to −0.14, *p* < 0.001) between relative HGS and fasting insulin (Figure 3(b)) with considerable heterogeneity (*τ* ^2^ = 0.006, *I* ^2^ = 87.8%, *H* ^2^ = 8.2, *Q* [3] = 30.706, *p* < 0.001).

#### 3.4.3. HOMA‐IR

Five [40, 41, 44–46] studies with five independent comparisons provided data from 6370 children and youth individuals to analyze the relationship between absolute HGS and HOMA‐IR. There was no significant correlation (*k* = 5; *c* = 5; *n* = 6370; Fisher’s *z* = 0.09; 95% CI = −0.04 to 0.22, *p* = 0.190) between absolute HGS and HOMA‐IR (Figure 2(c)) with considerable heterogeneity (*τ* ^2^ = 0.021, *I* ^2^ = 96.0%, *H* ^2^ = 25.1, *Q* [4] = 65.144, *p* < 0.001).

Six studies [35, 37, 40, 41, 44, 45] with six independent comparisons provided data from 6192 children and youth individuals to analyze the relationship between relative HGS and HOMA‐IR. There was a significant negative correlation (*k* = 6; *c* = 6; *n* = 6192; Fisher’s *z* = −0.25% CI = −0.28 to −0.15, *p* < 0.001) between relative HGS and HOMA‐IR (Figure 3(c)) with considerable heterogeneity (*τ* ^2^ = 0.004, *I* ^2^ = 80.0%, *H* ^2^ = 5.0, *Q* [5] = 34.849, *p* < 0.001).

### 3.5. Meta‐Analyses: HGS and Inflammatory Markers

The available data from the included studies (Table 2) allowed us to conduct correlation meta‐analyses polling Fisher’s *r*‐to‐*z* transformed correlation coefficients for relative HGS (relativized by body mass) and C‐reactive protein (CRP), high‐sensitive C‐reactive protein (hs‐CRP), complement components 3 (C3) and 4 (C4), and interleukin‐6 (IL‐6) (Table 3).

#### 3.5.1. CRP and hs‐CRP

Two studies [34, 38] with two independent comparisons provided data from 1142 children and youth individuals to analyze the relationship between relative HGS and CRP. There was a significant negative correlation (*k* = 2; *c* = 2; *n* = 1142; Fisher’s *z* = −0.18; 95% CI = −0.26 to −0.10, *p* < 0.001) between relative HGS and CRP (Figure 4(a)) with moderate heterogeneity (*τ* ^2^ = 0.001, *I* ^2^ = 44.7%, *H* ^2^ = 1.8, *Q* [1] = 1.810, *p* = 0.179).

Figure 4Association between relative HGS and inflammatory markers. (a) The top panel is HGS/BW and CRP, (b) the second panel is HGS/BW and hs‐CRP, (c) the third panel is HGS/BW and C3, (d) the fourth panel is HGS/BW and C4, and (e) the bottom panel represents HGS/BW and IL‐6.

**Figure:**
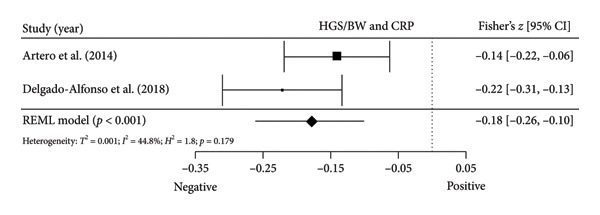
(a)

**Figure:**
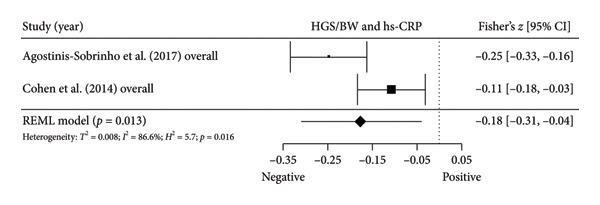
(b)

**Figure:**
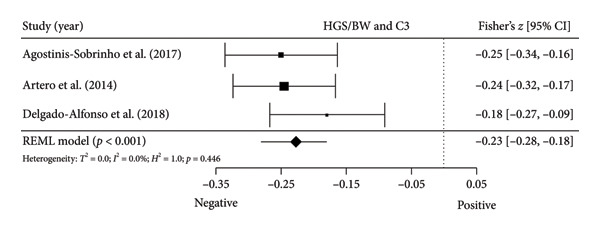
(c)

**Figure:**
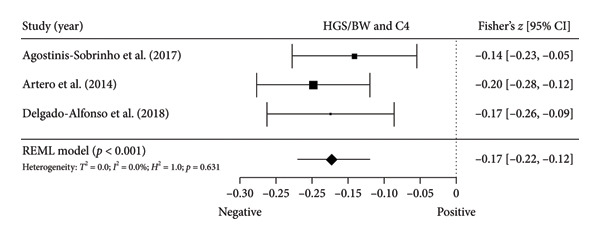
(d)

**Figure:**
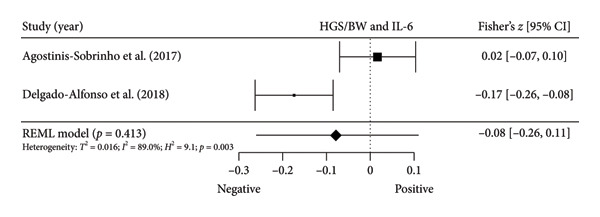
(e)

Two studies [33, 37] with two independent comparisons provided data from 1198 children and youth individuals to analyze the relationship between relative HGS and hs‐CRP. There was a significant negative correlation (*k* = 2; *c* = 2; *n* = 1198; Fisher’s *z* = −0.18; 95% CI = −0.31 to −0.04, *p* = 0.013) between relative HGS and hs‐CRP (Figure 4(b)) with considerable heterogeneity (*τ* ^2^ = 0.008, *I* ^2^ = 86.6%, *H* ^2^ = 5.7, *Q* [1] = 5.751, *p* = 0.016).

#### 3.5.2. Complement Components 3 (C3) and 4 (C4)

Three studies [33, 34, 38] with three independent comparisons provided data from 1671 children and youth individuals to analyze the relationship between relative HGS and C3 and C4. There was a significant negative correlation (*k* = 3; *c* = 3; *n* = 1671; Fisher’s *z* = −0.23; 95% CI = −0.28 to −0.18, *p* < 0.001) between relative HGS and C3 (Figure 4(c)) with low heterogeneity (*τ* ^2^ = 0.000, *I* ^2^ = 0.0%, *H* ^2^ = 1.0, *Q* [2] = 1.613, *p* = 0.446). There was also a significant negative correlation (*k* = 3; *c* = 3; *n* = 1671; Fisher’s *z* = −0.17; 95% CI = −0.22 to −0.12, *p* < 0.001) between relative HGS and C4 (Figure 4(d)) with low heterogeneity (*τ* ^2^ = 0.000, *I* ^2^ = 0.0%, *H* ^2^ = 1.0, *Q* [2] = 0.920, *p* = 0.631).

#### 3.5.3. IL‐6

Two studies [33, 38] with two independent comparisons provided data from 1032 children and youth individuals to analyze the relationship between relative HGS and IL‐6. There was no significant correlation (*k* = 2; *c* = 2; *n* = 1032; Fisher’s *z* = −0.08; 95% CI = −0.26 to 0.11, *p* = 0.413) between relative HGS and IL‐6 (Figure 4(e)) with considerable heterogeneity (*τ* ^2^ = 0.016, *I* ^2^ = 89.0%, *H* ^2^ = 9.1, *Q* [1] = 9.114, *p* = 0.003).

### 3.6. Sensitivity Analyses

A sensitivity analysis (the one study removed method) revealed that significant negative correlations between relative HGS and fasting insulin (all *p* < 0.001), HOMA‐IR (all *p* < 0.001), C3 (all *p* < 0.001), and C4 (all *p* < 0.001) remained after removing each one of the studies included in the main meta‐analyses (Supporting Information 3). In addition, a significant negative correlation between relative HGS and fasting glucose remained after removing Cohen et al.’s [37], Jiménez‐Pavón et al.’s [40], and López‐Gil et al.’s [44] studies but not after removing Jung et al. [41] study (Supporting Information 3). Sensitive analyses were not performed for correlations between relative HGS and h‐CRP or CRP because only two studies were included in their respective main meta‐analyses.

### 3.7. Subgroup Analyses

Due to the small number of included studies (*k* < 10), preplanned subgroup analyses were not performed to test whether the study design (cross‐sectional and longitudinal studies) would influence the results. Similarly, non‐preplanned subgroup analyses to examine whether participants’ sex would influence the results were not conducted for the same reason.

### 3.8. Publication Bias

Due to the small number of included studies, a visual analysis of the effect sizes (i.e., Fisher’s *r*‐to‐*z* transformed correlation coefficients) of the test results for resistance insulin and inflammatory markers did not indicate the presence or absence of publication bias (Supporting Information 4). As reported in the statistical analysis section, “Egger’s regression test” was not performed to assess the asymmetry of the funnel plot because the meta‐analyses involved less than 10 original studies [31].

### 3.9. Study Quality

All the cross‐sectional included studies (*n* = 12) [33–44] presented a good quality (> 75%) in the Joanna Briggs Critical Appraisal Tool. Most of the cross‐sectional included studies (66.7%, *n* = 8 of 12) [33, 36–42] reached 7 points (87.5%) in the Joanna Briggs Critical Appraisal Tool, while a quarter of the cross‐sectional included studies (*n* = 3 of 12) [35, 43, 44] reached the highest score possible (8 points, which correspond to 100%) (Supporting Information 5). All longitudinal included studies (*n* = 3) [45–47] reached the six points in the Joanna Briggs Critical Appraisal Tool, which correspond to 66.7% (moderate quality) of the highest possible score (9 points) (Supporting Information 6).

### 3.10. Certainty of Evidence (GRADE)

Because the present meta‐analysis included only cross‐sectional studies, the quality (level) rating of the evidence using GRADE started as low for all primary “outcomes” (Supporting Information 7). The evidence was not downgraded by one level for risk of bias (study quality) because all included studies presented a good study quality, and the evidence was not downgraded by one level for indirectness because the included studies investigated the same population (children/adolescents). However, for half of the primary outcomes, the evidence was downgraded by one level for inconsistency due to substantial/considerable statistical heterogeneity, and for most of the primary outcomes, the evidence was downgraded by one level for imprecision because the lower limit of the overall effect for the main analysis crossed the clinical threshold for relevance (*r* ≥ 0.1). For all primary outcomes, the evidence was downgraded by one level for publication bias, as visual analysis of the funnel plot did not indicate the presence or absence of publication bias. Therefore, the certainty of our estimates across primary outcomes was evaluated to be very low.

## 4. Discussion

Ample evidence suggests an inverse association between HGS and morbidity and mortality [1–9]. Research on the association between changes in HGS and risk factors for lifestyle‐related diseases in children and adolescents may be crucial for understanding the causal relationship behind the inverse association between HGS and morbidity/mortality [48]. This research aimed to systematically review and meta‐analyze the current evidence for the association between HGS and markers of insulin resistance and inflammation in children and adolescents. The meta‐analysis in this review used 11 of the 14 cross‐sectional studies. However, insufficient information in the longitudinal studies made it impossible to pool data for associations between HGS change and the changes in insulin resistance and inflammatory markers.

### 4.1. Findings From Longitudinal Studies

The results of this review indicate that no longitudinal studies with sufficient research information were found regarding the association between changes in HGS and changes in insulin resistance and inflammatory markers in children and adolescents. Therefore, we could not obtain evidence to elucidate the relationship between the two. However, several lifestyle‐related factors, such as sports type [15, 16], sleep duration [49], and diets [50–53], are reported to impact HGS or markers of insulin resistance and inflammation in children and adolescents. A study reported that HGS, when measured in childhood, was associated with prediabetes or Type 2 diabetes, similar to HGS measured in young adults and mid‐adulthood [54]. During the developmental period, HGS increases dramatically [55], and the changes in each individual are not uniform but show clear differences [56]. Furthermore, the results of the previous study described above [54] suggest that high HGS acquired during development favorably impacts markers of insulin resistance and may be associated with a lower incidence of diabetes. Therefore, future studies may elucidate the association between changes in HGS and changes in insulin resistance and inflammatory markers in children and adolescents.

### 4.2. Findings From Cross‐Sectional Studies

The results from cross‐sectional studies indicated that the association between HGS and several markers of insulin resistance and inflammation existed when studies utilized the relative HGS (Figures 3 and 4). However, no significant relationship was found when studies used absolute HGS (Figure 2). The results from each study were adjusted for different factors such as age [33–35, 37, 41, 44–46], sex [33–35, 37, 38, 41, 44–46], pubertal or maturation stage [33–35, 37, 38, 40], physical activity [37], BMI or BMI‐standard deviation score [38, 40, 41, 46], school type [45], and family background [45, 46]. Unlike longitudinal studies that observe changes in HGS, participants’ HGS in cross‐sectional studies may be determined by complex factors, including being influenced by genetics [10] and nongenetic factors [11], including in utero [57, 58]. The same may apply to insulin resistance and inflammatory markers. At least a couple of possibilities exist for explaining the observed differences in the association of absolute and relative HGS with insulin resistance and inflammation markers.

Firstly, physical and sporting activities may be a possible factor. It is well known that physical and sporting activities impact insulin resistance and inflammatory markers [59, 60] and HGS [15]. In addition, physical and sporting activities also control the accumulation of excess body fat, and there is an inverse association between accelerometer‐measured physical activity and body fat mass in adolescents [61]. A study compared the HGS of children divided into two groups based on recommended physical activity guidelines (> 60 min of moderate and vigorous physical activity per day) and found that HGS was similar between the two groups [62]. Even if daily physical activity does not influence HGS in children and adolescents who meet the recommended criteria for physical activity guidelines [62, 63], physical activity‐induced changes in body fat mass may affect body mass and relative HGS per body mass. On the other hand, the type of sports or physical activity (gripping tools with the hands or not) may influence the improvement of HGS in developing children and adolescents [15–17]. However, it remains unclear how the type of sports or physical activity affects insulin resistance and inflammatory markers. Considering that differences in sport type (gripping tools with hands) may influence the improvement of HGS and body composition in children and adolescents, investigating their effects on insulin resistance and inflammatory markers may help to understand the current study results.

The quality and quantity of diets may be another factor influencing the association between absolute/relative HGS and insulin resistance and inflammatory markers. Only two of the 15 studies [33, 46] included in this systematic review used diet‐related variables as covariates. Poor dietary conditions may affect low HGS in children and adolescents [50, 51]. A study reported the possible association of higher culinary preparation intake with higher HGS and that of high ultra‐processed food with lower HGS in male teenagers [51]. Longitudinal follow‐up studies reported a possibility that there is an association between the high consumption of ultra‐processed foods and greater whole‐body and abdominal adiposity [64]. Therefore, future research should consider the dietary intake of participants when analyzing the results. Furthermore, it is known that insulin resistance and CRP are associated with excess body fat [65, 66], and overweight and obesity are causes of low relative HGS. Studies included in this review reported that the proportion of children and adolescents who were overweight/obese was approximately 10%–20% [34, 35, 37, 40]. In contrast, no studies have controlled for the impact of body fat mass or percentage. Therefore, it remains possible that the influence of accumulated body fat on the reported results has yet to be excluded entirely.

Lastly, the significance of relative HGS (i.e., divided by body mass), rather than the absolute value, being associated with insulin resistance and inflammatory markers is unclear. Therefore, it is not concluded from the meta‐analysis results that high HGS is associated with desirable insulin resistance and inflammatory markers. The use of a ratio (HGS/body mass) can sometimes lead to spurious results [67]. This might explain the discrepancy between absolute and relative HGS. Many include relatives because they are trying to “control” the denominator. However, this assumes that the numerator is scaled the same across all levels of the denominator. In order to elucidate the relationship between the two, it may be necessary to investigate the association between changes in HGS and marker changes in insulin resistance and inflammation in children and adolescents, rather than cross‐sectional studies. Future research is needed to resolve this issue.

### 4.3. Strengths and Limitations

To our knowledge, this is the first meta‐analysis to provide an association between HGS and insulin resistance and inflammatory markers in children and adolescents. This review revealed that no longitudinal studies reported an association between the two. On the other hand, the cross‐sectional studies used in the meta‐analysis included studies with relatively large sample sizes. There are some limitations that need to be discussed. First, our meta‐analysis results utilize results from cross‐sectional studies, which limits the ability to determine the causal association between HGS and insulin resistance and inflammatory markers. Longitudinal follow‐up research is needed to resolve these issues. Second, some studies have used BMI or pubertal status as surrogate markers of body fatness. Therefore, the results may differ when using the directly estimated body fat mass (or percentage) that impacts insulin resistance and inflammatory markers. Third, some included studies reported crude *r* values, while others adjusted *r* values for sex and age and/or pubertal stage and body mass index; therefore, our results may be biased due to confounding factors. Fourth, diet quality and balance may influence HGS and markers of insulin resistance and inflammation, but almost no studies have controlled these effects. Fifth, most of the included studies in this review utilized HOMA‐IR as an indicator of hepatic insulin resistance, with one study each utilizing QUICKI [40] (a surrogate for glucose clamp‐derived measures of insulin sensitivity) and oral glucose tolerance test (OGTT) [43], both of which most reflect peripheral (e.g., skeletal muscle) insulin resistance. One study [43] reported that the OGTT had a stronger relationship with relative HGS than the HOMA‐IR, which may be helpful in future studies. Furthermore, it may be necessary to investigate the relationship with markers of protective effect against tissue inflammation. Sixth, some cross‐sectional studies included in this systematic review were not included in the meta‐analysis due to insufficient information, and therefore, we strongly recommend future studies also report *r* values for all correlation analyses. Seventh, due to the small number of studies included in each meta‐analysis, we were unable to investigate the factors that could explain the significant statistical heterogeneity found for many analyses regarding relative HGS and insulin resistance and inflammatory markers. We were also unable to identify the presence or absence of publication bias due to the small number of studies included in each meta‐analysis. Finally, the certainty of our estimates across primary outcomes was evaluated to be very low, which indicates a very uncertainty about the estimate. Thus, our results should be interpreted cautiously.

## 5. Conclusion

The results from cross‐sectional studies indicated the association (very low evidence) between HGS and several markers of insulin resistance and inflammation existed when studies utilized the relative HGS per body mass. However, no significant relationship was found when studies used absolute HGS. The reason for the difference in the results between absolute and relative HGS is unknown, but lifestyle‐related factors that affect HGS and insulin resistance and inflammatory markers may be involved, and these may not be adequately controlled. Furthermore, as longitudinal studies were limited and did not include enough results to conduct a meta‐analysis, future longitudinal follow‐up studies are an important means of resolving these issues.

**Nomenclature**
BMI: Body mass index
C3: Complement component 3
C4: Complement component 4
CRP: C‐reactive protein
GRADE: Certainty of evidence
HGS: Handgrip strength
HOMA‐IR: Homeostatic model assessment for insulin resistance
hs‐CRP: High‐sensitive C‐reactive protein
IL‐6: Interleukin‐6
OGTT: Oral glucose tolerance test
QUICKI: Quantitative insulin sensitivity check index

## Disclosure

A preprint has previously been published in Research Square [68].

## Conflicts of Interest

The authors declare no conflicts of interest.

## Funding

This study was supported by the Japan Society for the Promotion of Science, Grants‐in‐Aid for Scientific Research (Grant Number: 22K11610), and the Institute of Health and Sports Science & Medicine, Juntendo University.

## Supporting information


**Supporting Information** Additional supporting information (Supporting Information) to this article can be found in the Supporting Information section.

## Data Availability

The data that support the findings of this study are available from the corresponding author upon reasonable request.
