# Supplementary material for: Associations Between Handgrip Strength and Markers of Insulin Resistance and Inflammation in Childhood and Adolescence: A Systematic Review With Meta‐Analysis
Source: Transl Sports Med. 2026 Jan 5;2026:1091342. doi: 10.1155/tsm2/1091342 (PMC12782331; doi:10.1155/tsm2/1091342)
Supplement: Supplementary file 1 — Supporting Information Additional supporting information (Supporting Information) to this article can be found in the Supporting Information section. [file TSM2-2026-1091342-s001.pdf]

Supplementary Material Description

This supplementary material provides additional information and data related to the study presented in the main manuscript. It includes:

Supplementary Material 1

Complete search strategy.

Supplementary Material 2

Reasons for inclusion (green) and exclusion (red) after second screening (full-text reading).

Supplementary Material 3

Synthesis of all sensitivity analyses (the one study removed method).

Supplementary Material 4

Funnel plot of Fisher's r-to-z transformed correlation coefficient effect sizes within the studies included in the meta-analyses.

Supplementary Material 5

The methodological quality of the included cross-sectional studies was assessed using the Joanna Briggs Critical Appraisal Tool.

Supplementary Material 6

The methodological quality of the included longitudinal studies was assessed using the Joanna Briggs Critical Appraisal Tool.

Supplementary Material 7

Summary of certainty assessment by the Grading of Recommendations, Assessment, Development, and Evaluation (GRADE) method.

Supplementary Material 1. Complete search strategy.

MEDLINE

All fields:

((handgrip strength OR grip strength OR grip) AND (resistin OR insulin resistance OR insulin sensitivity OR inflammation OR cytokines OR acute phase proteins)) AND (child OR children OR ten OR teenager OR pediatric OR adolescents OR adolescence OR juvenile)

Scopus

All fields:

(ALL (handgrip AND strength OR grip AND strength OR grip) AND ALL (resistin OR insulin AND resistance OR insulin AND sensitivity OR inflammation OR cytokines OR acute AND phase AND proteins) AND ALL (child OR children OR ten OR teenager OR pediatric OR adolescents OR adolescence OR juvenile))

Web of Science

All fields:

handgrip strength OR grip strength OR grip (All Fields) AND resistin OR insulin resistance OR insulin sensitivity OR inflammation OR cytokines OR acute phase proteins (All Fields) AND child OR children OR ten OR teenager OR pediatric OR adolescents OR adolescence OR juvenile (All Fields)

Embase

('handgrip strength' OR 'grip strength'/exp OR grip) AND (resistin/exp OR 'insulin resistance'/exp OR 'insulin sensitivity'/exp OR inflammation/exp OR cytokine/exp OR 'acute phase protein'/exp) AND (child/exp OR children OR ten OR teenager OR pediatric/exp OR adolescent/exp OR adolescence/exp OR juvenile/exp)

Cochrane Central Register of Controlled Trials (CENTRAL)

All Text:

(handgrip strength OR grip strength OR grip) AND (resistin OR insulin resistance OR insulin sensitivity OR inflammation OR cytokines OR acute phase proteins) AND (child OR children OR ten OR teenager OR pediatric OR adolescents OR adolescence OR juvenile)

Supplementary Material 2. Reasons for inclusion (green) and exclusion (red) after second screening (full-text reading).

Study	Reasons	Situation
Agostinis-Sobrinho et al. (2017)[1]	OK. Data available.	Included in the systematic review and meta-analysis
Artero et al. (2011)[2]	OK. Data available.	Included in the systematic review and meta-analysis
Artero et al. (2014)[3]	OK. Data available.	Included in the systematic review and meta-analysis
Castro-Piñero et al. (2019)[3]	OK. Additional data was requested from the corresponding author. The author provided *r* values.	Included in the systematic review and meta-analysis
Cohen et al. (2014)[4]	OK. Data available.	Included in the systematic review and meta-analysis
de Lima et al. (2021a)[5]	Wrong population (14 to 19 years old).	Excluded.
de Lima et al. (2021b)[6]	Wrong population (14 to 19 years old).	Excluded.
de Lima et al. (2024)[7]	Wrong population (14 to 19 years old).	Excluded.
Delgado-Alfonso et al. (2018)[8]	OK. Data available.	Included in the systematic review and meta-analysis

Demmer et al. (2016)[9]	OK. Longitudinal study.	Included in the systematic review.
Haapala et al. (2023)[10]	OK. Additional data was requested from the corresponding author. We have no answer. Although were able to estimate *r* values from standardized beta coefficients, the handgrip strength was relativized by participants lean mass and, therefore, it was not possible to include this study either in the meta-analysis about absolute or relativized (by participants body mass) handgrip strength.	Included in the systematic review.
Jimenez-Pavon et al. (2012)[11]	OK. Data available.	Included in the systematic review and meta-analysis
Jung et al. (2022)[12]	OK. The authors did not report if beta coefficients were standardized or unstandardized. Additional data was requested from the corresponding author. The author provided *r* values.	Included in the systematic review and meta-analysis
Lang et al. (2019)[13]	OK. Additional data was requested from the corresponding author. Answer: "*Ive thought about your request. The paper you referred to only included cycles 1 and 2 of the Canadian Health Measures Survey. We now have data from cycles 5 and 6 (2017 to 2019). This is an additional ~4000 kids spanning four cross-sectional timepoints. For your request, I would have to re-run everything from scratch. The original analysis I did is dated now and wouldn't be worth digging up. To do a good job with this it would take me several hours – potentially a full day of work. With how the survey works, there are several datafiles that need to be merged and cleaned. There are lots of details that need to be cared for to ensure mistakes aren't made. To be honest, I have lots of commitments*	Included in the systematic review.

	on my plate right now – just like everyone else in our chosen career as researchers 😊. *We all know how work can pile up if we constantly say yes to every request. Im finding it hard to justify putting the time in with little benefit. I do feel selfish saying this and what to emphasize that im not a selfish person! It's a tough sell to my manager as well. It's an interesting meta-analysis and important to do! Im very interested in this research area and have done a lot of work to date. Unfortunately, im going to have to decline your request and wish you the best of luck with your important research."* To note, it was not possible to convert unstandardized beta coefficients to standardized beta coefficients and, consequently, *r* values.	
Li et al. (2018)[14]	Additional data was requested from the corresponding author. Answer: "*Dear Dr. xxxx, Thank you [for reaching out. I'm terribly sorry that I won't be able to help with the data you requested - my old computer died on me before I had a chance to copy data from it.*" The corresponding author were emailed again to confirm if beta coefficients were standardized or unstandardized. Answer: "*The betas were unstandardized."* However, we were not able to estimate *r* values from unstandardized beta coefficients because there was no information regarding standard deviation of relative handgrip strength (kg per kg of body weight).	Included in the systematic review
López-Gil et al. (2021)[15]	OK. Additional data was requested from the corresponding author. The author provided *r* values.	Included in the systematic review and meta-analysis
Ruiz et al. (2008)[16]	Additional data was requested from the corresponding author. Answer: "*Sorry!! I have no access to the data :-(*".	Excluded

Tarp et al. (2019)[17]	OK. Additional data was requested from the corresponding author. The author provided r values.	Included in the systematic review and meta-analysis
Zaqout et al. (2016)[18]	OK. Additional data was requested from the corresponding author. We have no answer. However, we were able to estimate *r* values from standardized beta coefficients.	Included in the systematic review and meta-analysis

1. Agostinis-Sobrinho CA, Moreira C, Abreu S, Lopes L, Sardinha LB, Oliveira-Santos J, et al. Muscular fitness and metabolic and inflammatory biomarkers in adolescents: results from LabMed physical activity study. Scand J Med Sci Sport. 2017;27:1873–80.

2. Artero EG, Ruiz JR, Ortega FB, España-Romero V, Vicente-Rodríguez G, Molnar D, et al. Muscular and cardiorespiratory fitness are independently associated with metabolic risk in adolescents: The HELENA study. Pediatr Diabetes. 2011;12:704–12.

3. Castro-Piñero J, Laurson KR, Artero EG, Ortega FB, Labayen I, Ruperez AI, et al. Muscle strength field-based tests to identify European adolescents at risk of metabolic syndrome: The HELENA study. J Sci Med Sport. 2019;22:929–34.

4. Cohen DD, Gómez-Arbeláez D, Camacho PA, Pinzon S, Hormiga C, Trejos-Suarez J, et al. Low muscle strength is associated with metabolic risk factors in Colombian children: The ACFIES study. PLoS One. 2014;9:1–10.

5. de Lima TR, Sui X, de Lima LRA, Silva DAS. Muscle strength and its association with cardiometabolic variables in adolescents: does the expression of muscle strength values matter? World J Pediatr. Springer Singapore; 2021;17:597–608.

6. de Lima TR, Sui X, Silva DAS. Normalization of muscle strength measurements in the assessment of cardiometabolic risk factors in adolescents. Int J Environ Res Public Health. 2021;18:8428.

7. de Lima TR, Silva DAS. Muscle strength indexes and its association with cardiometabolic risk factors in adolescents: an allometric approach. Res Q Exerc Sport. 2024;95:289–302.

8. Delgado-Alfonso A, Pérez-Bey A, Conde-Caveda J, Izquierdo-Gómez R, Esteban-Cornejo I, Gómez-Martínez S, et al. Independent and combined associations of physical fitness components with inflammatory biomarkers in children and adolescents. Pediatr Res. Springer US;

2018;84:704–12.
9. Demmer DL, Beilin LJ, Hands B, Burrows S, Cox KL, Straker LM, et al. Effects ofmuscle strength and endurance on blood pressure and related cardiometabolic risk factors from childhood to adolescence. J Hypertens. 2016;34:2365–75.
10. Haapala EA, Kuronen E, Ihalainen JK, Lintu N, Leppänen MH, Tompuri T, et al. Cross-sectional associations between physical fitness and biomarkers of inflammation in children—The PANIC study. Scand J Med Sci Sport. 2023;33:1000–9.
11. Jiménez-Pavón D, Ortega FB, Valtueña J, Castro-Piñero J, Gómez-Martínez S, Zaccaria M, et al. Muscular strength and markers of insulin resistance in European adolescents: The HELENA Study. Eur J Appl Physiol. 2012;112:2455–65.
12. Jung HW, Lee J, Kim J. Handgrip strength is associated with metabolic syndrome and insulin resistance in children and adolescents: analysis of Korea national health and nutrition examination survey 2014-2018. J Obes Metab Syndr. 2022;31:334–44.
13. Lang JJ, Larouche R, Tremblay MS. The association between physical fitness and health in a nationally representative sample of Canadian children and youth aged 6 to 17 years. Heal Promot Chronic Dis Prev Canada. 2019;39:104–11.
14. Li S, Zhang R, Pan G, Zheng L, Li C. Handgrip strength is associated with insulin resistance and glucose metabolism in adolescents: evidence from national health and nutrition examination survey 2011 to 2014. Pediatr Diabetes. 2018;19:375–80.
15. López-Gil JF, Weisstaub G, Ramírez-Vélez R, García-Hermoso A. Handgrip strength cut-off points for early detection of cardiometabolic risk in Chilean children. Eur J Pediatr. 2021;180:3483–9.
16. Ruiz JR, Ortega FB, Wärnberg J, Moreno LA, Carrero JJ, Gonzalez-Gross M, et al. Inflammatory proteins and muscle strength in adolescents: the AVENA study. Arch Pediatr Adolesc Med. 2008;162:462.
17. Tarp J, Bugge A, Møller NC, Klakk H, Rexen CT, Grøntved A, et al. Muscle fitness changes during childhood associates with improvements in cardiometabolic risk factors: a prospective study. J Phys Act Health. 2019;16:108–15.
18. Zaqout M, Michels N, Bammann K, Ahrens W, Sprengeler O, Molnar D, et al. Influence of physical fitness on cardio-metabolic risk factors in European children. The IDEFICS study. Int J Obes. Nature Publishing Group; 2016;40:1119–25.

Supplementary Material 3. Synthesis of all sensitivity analyses (the one study removed method).

A. Sensitivity analyses for the correlation between relative handgrip strength and fasting glucose (n = 4).

Removing…	Test for overall effect	Heterogeneity
Cohen et al. (2014)	Z = –0.07 [–0.11 to –0.02]; P = 0.002	T^2 = 0.0005; H^2 = 1.6; I^2 = 35.8%; P = 0.153
Jiménez-Pavón et al. (2012)	Z = –0.07 [–0.11 to –0.03]; P < 0.001	T^2 = 0.0003; H^2 = 1.3; I^2 = 20.6%; P = 0.182
Jung et al. (2022)	Z = –0.06 [–0.12 to 0.01]; P = 0.073	T^2 = 0.002; H^2 = 2.2; I^2 = 54.4%; P = 0.116
López-Gil et al. (2021)	Z = –0.05 [–0.08 to –0.02]; P < 0.001	T^2 = 0.000; H^2 = 1.0; I^2 = 0%; P = 0.568

B. Sensitivity analyses for the correlation between relative handgrip strength and fasting insulin (n = 4).

Removing…	Test for overall effect	Heterogeneity
Castro-Piñero et al. (2019)	Z = –0.25 [–0.35 to –0.14]; P < 0.001	T^2 = 0.008; H^2 = 9.3; I^2 = 89.2%; P < 0.001
Jiménez-Pavón et al. (2012)	Z = –0.23 [–0.35 to –0.11]; P < 0.001	T^2 = 0.010; H^2 = 11.5; I^2 = 91.3%; P < 0.001
Jung et al. (2022)	Z = –0.19 [–0.24 to –0.15]; P < 0.001	T^2 = 0.0004; H^2 = 1.3; I^2 = 25.0%; P = 0.280
López-Gil et al. (2021)	Z = –0.25 [–0.35 to –0.15]; P < 0.001	T^2 = 0.007; H^2 = 10.4; I^2 = 90.4%; P < 0.001

C. Sensitivity analyses for the correlation between relative handgrip strength and HOMA-IR (n = 6).

Removing…	Test for overall effect	Heterogeneity
Artero et al. (2011)	Z = –0.22 [–0.29 to –0.15]; P < 0.001	T^2 = 0.005; H^2 = 5.9; I^2 = 83.2%; P < 0.001
Cohen et al. (2014)	Z = –0.22 [–0.29 to –0.15]; P < 0.001	T^2 = 0.005; H^2 = 5.8; I^2 = 82.9%; P < 0.001
Jiménez-Pavón et al. (2012)	Z = –0.21 [–0.28 to –0.14]; P < 0.001	T^2 = 0.006; H^2 = 5.7; I^2 = 82.5%; P < 0.001

Jung et al. (2022)	Z = –0.19 [–0.23 to –0.16]; P < 0.001	T^2 = 0.00002; H^2 = 1.0; I^2 = 1.5%; P = 0.493
López-Gil et al. (2021)	Z = –0.22 [–0.29 to –0.15]; P < 0.001	T^2 = 0.005; H^2 = 6.1; I^2 = 83.7%; P < 0.001
Tarp et al. (2019)	Z = –0.23 [–0.29 to –0.17]; P < 0.001	T^2 = 0.004; H^2 = 5.0; I^2 = 80.0%; P < 0.001

D. Sensitivity analyses for the correlation between relative handgrip strength and complement component 3 (n = 3).

Removing…	Test for overall effect	Heterogeneity
Agostinis-Sobrinho et al. (2017)	Z = –0.22 [–0.28 to –0.15]; P < 0.001	T^2 = 0.0004; H^2 = 1.2; I^2 = 16.8%; P = 0.273
Artero et al. (2014)	Z = –0.22 [–0.28 to –0.15]; P < 0.001	T^2 = 0.0005; H^2 = 1.3; I^2 = 21.4%; P = 0.259
Delgado-Alfonso et al. (2018)	Z = –0.25 [–0.31 to –0.19]; P < 0.001	T^2 = 0.000; H^2 = 0.0; I^2 = 0%; P = 0.933

E. Sensitivity analyses for the correlation between relative handgrip strength and complement component 4 (n = 3).

Removing…	Test for overall effect	Heterogeneity
Agostinis-Sobrinho et al. (2017)	Z = –0.19 [–0.25 to –0.13]; P < 0.001	T^2 = 0.000; H^2 = 0.0; I^2 = 0%; P = 0.690
Artero et al. (2014)	Z = –0.16 [–0.22 to –0.10]; P < 0.001	T^2 = 0.000; H^2 = 0.0; I^2 = 0%; P = 0.600
Delgado-Alfonso et al. (2018)	Z = –0.17 [–0.23 to –0.11]; P < 0.001	T^2 = 0.000; H^2 = 0.0; I^2 = 0%; P = 0.338

Supplementary Material 4. Funnel plot of Fisher's r-to-z transformed correlation coefficient effect sizes within the studies included in the meta-analyses.

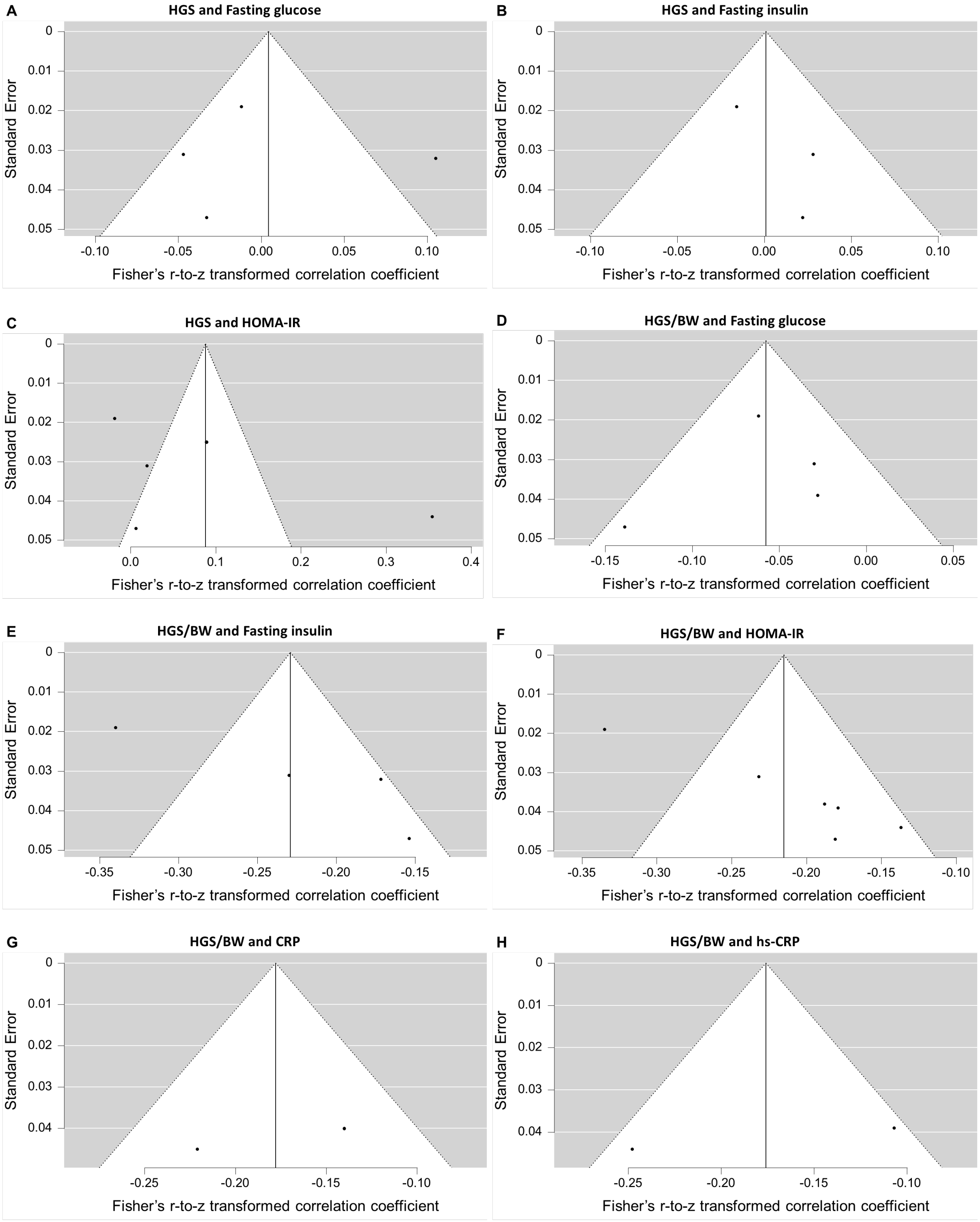

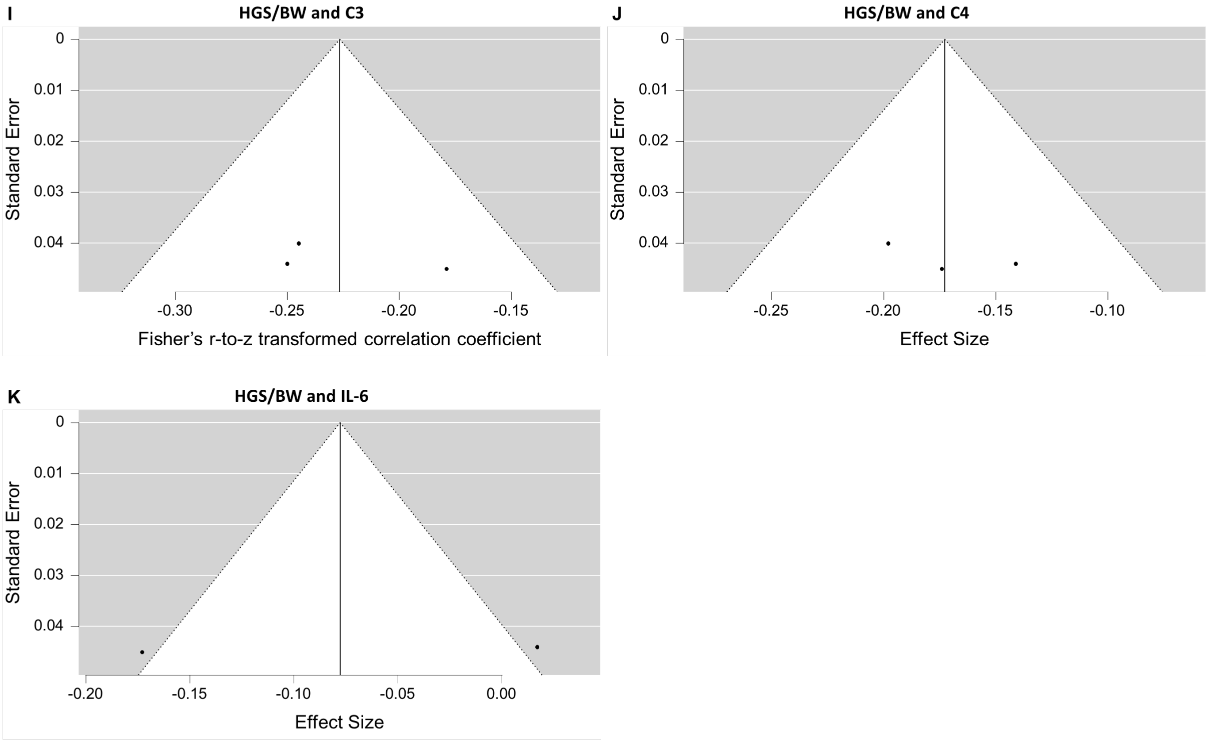

Legends: HGS: handgrip strength. HGS/BW: handgrip strength relativized by participants body weight. HOMA-IR: homeostasis model assessment-estimated insulin resistance. QUICKI: quantitative insulin sensitivity check index. CRP: C-reactive protein. hs-CRP: high-sensitivity C-reactive protein. C3: complement component 3. C4: complement component 4. IL-6: interleukin-6.

Supplementary Material 5. The methodological quality of the included cross-sectional studies was assessed using the Joanna Briggs Critical Appraisal Tool.

Studies	1.Were the criteria for inclusion in the sample clearly defined?	2.Were the study subjects and the setting described in detail?	3. Was the exposure measured in a valid and reliable way?	4. Were objective, standard criteria used for measurement of the condition?	5. Were confoundin g factors identified?	6. Were strategies to deal with confounding factors stated?	7. Were the outcomes measured in a valid and reliable way?	8. Was appropriate statistical analysis used?
Agostinis-Sobrinho et al. (2017)	Unclear	Yes	Yes	Yes	Yes	Yes	Yes	Yes
Artero et al. (2011)	Yes	Yes	Yes	Yes	Yes	Yes	Yes	Yes
Artero et al. (2014)	Unclear	Unclear	Yes	Yes	Yes	Yes	Yes	Yes
Castro-Piñero et al. (2019)	Unclear	Yes	Yes	Yes	Yes	Yes	Yes	Yes
Cohen et al. (2014)	Unclear	Yes	Yes	Yes	Yes	Yes	Yes	Yes
Delgado-Alfonso et al. (2018)	Unclear	Yes	Yes	Yes	Yes	Yes	Yes	Yes
Haapala et al. (2023)	Unclear	Yes	Yes	Yes	Yes	Yes	Yes	Yes
Jimenez-Pavon et al. (2012)	Unclear	Yes	Yes	Yes	Yes	Yes	Yes	Yes
Jung et al. (2022)	Unclear	Yes	Yes	Yes	Yes	Yes	Yes	Yes
Lang et al. (2019)	Unclear	Yes	Yes	Yes	Yes	Yes	Yes	Yes
Li et al. (2018)	Yes	Yes	Yes	Yes	Yes	Yes	Yes	Yes
López-Gil et al. (2021)	Yes	Yes	Yes	Yes	Yes	Yes	Yes	Yes

Supplementary Material 6. The methodological quality of the included longitudinal studies was assessed using the Joanna Briggs Critical Appraisal Tool.

Studies	1. Were the two groups similar and recruited from the same population?	2. Were the exposures measured similarly to assign people to both exposed and unexposed groups?	3. Was the exposure measured in a valid and reliable way?	4. Were confounding factors identified?	5. Were strategies to deal with confounding factors stated?	6. Were the groups/participants free of the outcome at the start of the study (or at the moment of exposure)?	7. Were the outcomes measured in a valid and reliable way?	8. Was the follow up time reported and sufficient to be long enough for outcomes to occur?	9. Was follow up complete, and if not, were the reasons to loss to follow up described and explored?	10. Were strategies to address incomplete follow up utilized?	11. Was appropriate statistical analysis used?
Demmer et al. (2016)	Not applicable	Not applicable	Yes	Yes	Yes	Unclear	Yes	Yes	No	Unclear	Yes
Tarp et al. (2019)	Not applicable	Not applicable	Yes	Yes	Yes	Unclear	Yes	Yes	No	Unclear	Yes
Zaqout et al. (2016)	Not applicable	Not applicable	Yes	Yes	Yes	Unclear	Yes	Yes	No	Unclear	Yes

Supplementary Material 7. Summary of certainty assessment by the Grading of Recommendations, Assessment, Development, and Evaluation (GRADE) method.

Certainty Assessment								
Outcomes (correlation)	Participants (studies)	Risk of bias (study quality)	Inconsistency	Indirectness	Imprecision	Publication bias	Overall certainty	Overall effect [95%CI]
HGS × Fasting glucose	5252 (4 cross-sectional)[a]	Not serious[b]	Serious[c]	Not serious[e]	Serious[f]	Not possible to determine[g]	⊕○○○	*r*: 0.004 [−0.06 to 0.07]
HGS × Fasting insulin	4302 (3 cross-sectional)[a]	Not serious[b]	Not serious[d]	Not serious[e]	Serious[f]	Not possible to determine[g]	⊕○○○	*r*: 0.0008 [−0.03 to 0.03]
HGS × HOMA-IR	6370 (5 cross-sectional)[a]	Not serious[b]	Serious[c]	Not serious[e]	Serious[f]	Not possible to determine[g]	⊕○○○	*r*: 0.09 [−0.04 to 0.22]
HGS/BW × Fasting glucose	4971 (4 cross-sectional)[a]	Not serious[b]	Not serious[d]	Not serious[e]	Serious[f]	Not possible to determine[g]	⊕○○○	*r*: −0.06 [−0.09 to −0.03]
HGS/BW × Fasting insulin	5252 (4 cross-sectional)[a]	Not serious[b]	Serious[c]	Not serious[e]	Not serious	Not possible to determine[g]	⊕○○○	*r*: −0.23 [−0.30 to −0.14]
HGS/BW × HOMA-IR	6192 (6 cross-sectional)[a]	Not serious[b]	Serious[c]	Not serious[e]	Not serious	Not possible to determine[g]	⊕○○○	*r*: −0.22 [−0.27 to −0.15]
HGS/BW × CRP	1142 (2 cross-sectional)[a]	Not serious[b]	Not serious[d]	Not serious[e]	Not serious	Not possible to determine[g]	⊕○○○	*r*: −0.18 [−0.25 to −0.10]
HGS/BW × hs-CRP	1198 (2 cross-sectional)[a]	Not serious[b]	Serious[c]	Not serious[e]	Serious[f]	Not possible to determine[g]	⊕○○○	*r*: −0.18 [−0.30 to −0.04]
HGS/BW × C3	1671 (3 cross-sectional)[a]	Not serious[b]	Not serious[d]	Not serious[e]	Not serious	Not possible to determine[g]	⊕○○○	*r*: −0.23 [−0.27 to −0.18]

HGS/BW × C4	1671<br>(3 cross-sectional)[a]	Not serious[b]	Not serious[d]	Not serious[e]	Not serious	Not possible to determine[g]	⊕◯◯◯	*r*: −0.17 [−0.22 to −0.12]
HGS/BW × IL-6	1032<br>(2 cross-sectional)[a]	Not serious[b]	Serious[c]	Not serious[e]	Serious[f]	Not possible to determine[g]	⊕◯◯◯	*r*: −0.08 [−0.25 to 0.11]

r: correlation coefficient. CI: confidence interval. HGS: handgrip strength. HGS/BW: handgrip strength relativized by participants body weight. HOMA-IR: homeostasis model assessment-estimated insulin resistance. CRP: C-reactive protein. hs-CRP: high-sensitivity C-reactive protein. C3: complement component 3. C4: complement component 4. IL-6: interleukin-6.

Notes:

[a]Certainty started as “Low” because observational (cross-sectional) studies were included in the analyses.

[b]Not downgraded because the included studies presented a low risk of bias.

[c]Downgraded because there was substantial/considerable statistical heterogeneity.

[d]Not downgraded because there was no substantial/considerable statistical heterogeneity.

[e]Not downgraded because the included studies investigated the same population (children/adolescents).

[f]Downgraded because the low limit of the overall effect crossed the clinical threshold for relevance ($r \geq 0.10$) (Pearson thresholds [17]).

[g]Downgraded because visual analysis of the of funnel plot was not able to indicate presence or absence of publication bias.

References

17. Munro, B. H. (1986). *Statistical methods for health care research*. JB Lippincott.
